# ncRNA orthologies in the vertebrate lineage

**DOI:** 10.1093/database/bav127

**Published:** 2016-03-15

**Authors:** Miguel Pignatelli, Albert J. Vilella, Matthieu Muffato, Leo Gordon, Simon White, Paul Flicek, Javier Herrero

**Affiliations:** 1 European Molecular Biology Laboratory, European Bioinformatics Institute; 2 Wellcome Trust Sanger Institute, Wellcome Trust Genome Campus, Hinxton, Cambridge CB10 1SD, UK; 3 UCL Cancer Institute, University College London, London WC1E 6BT, UK

## Abstract

Annotation of orthologous and paralogous genes is necessary for many aspects of evolutionary analysis. Methods to infer these homology relationships have traditionally focused on protein-coding genes and evolutionary models used by these methods normally assume the positions in the protein evolve independently. However, as our appreciation for the roles of non-coding RNA genes has increased, consistently annotated sets of orthologous and paralogous ncRNA genes are increasingly needed. At the same time, methods such as PHASE or RAxML have implemented substitution models that consider pairs of sites to enable proper modelling of the loops and other features of RNA secondary structure. Here, we present a comprehensive analysis pipeline for the automatic detection of orthologues and paralogues for ncRNA genes. We focus on gene families represented in Rfam and for which a specific covariance model is provided. For each family ncRNA genes found in all Ensembl species are aligned using Infernal, and several trees are built using different substitution models. In parallel, a genomic alignment that includes the ncRNA genes and their flanking sequence regions is built with PRANK. This alignment is used to create two additional phylogenetic trees using the neighbour-joining (NJ) and maximum-likelihood (ML) methods. The trees arising from both the ncRNA and genomic alignments are merged using TreeBeST, which reconciles them with the species tree in order to identify speciation and duplication events. The final tree is used to infer the orthologues and paralogues following Fitch's definition. We also determine gene gain and loss events for each family using CAFE. All data are accessible through the Ensembl Comparative Genomics (‘Compara’) API, on our FTP site and are fully integrated in the Ensembl genome browser, where they can be accessed in a user-friendly manner.

**Database URL:** http://www.ensembl.org

## Introduction

Non-coding RNAs (ncRNAs) are RNA molecules that are not translated into proteins. Although the actual number of ncRNAs in eukaryotic genomes remains unknown, estimates range in thousands (1, 2). Our view of RNA biology has been revolutionized by the discovery and characterisation of the various roles that ncRNA plays in central biological processes such as splicing (3), genome defense (4, 5), chromosome structure (6, 7) and the regulation of gene expression (8). ncRNAs have also been linked to human diseases including cancer (9, 10), neurological disorders such as Parkinson's (11) and Alzheimer's disease (12–14), cardiovascular disorders (15, 16) and numerous others [for a complete review see (17)]. ncRNAs are now acknowledged as crucial components of cellular and organismal complexity (18) and the correct characterization of ncRNA content is increasingly important for genome annotation (19–21).

As opposed to long ncRNAs, the vast majority of short ncRNA are fewer than 200 bp in length and lack many signatures of mRNAs, including 5' capping, splicing and poly-adenylation (22). The best known small ncRNAs include ribosomal RNA (rRNA), tRNA, snoRNA (23), piwiRNAs (24), riboswitches (25), snRNAs (26) and microRNAs (miRNAs) (27).

Among the most abundant ncRNA classes in mammalian genome are miRNAs and snoRNAs. In animals these miRNA molecules mediate post-transcriptional gene silencing by influencing the translation of mRNA into proteins (28, 29) and are the most widely studied class of ncRNA to date. miRNAs are estimated to regulate the translation of > 60% of protein-coding genes (30, 31). By this mechanism they are directly involved in regulating many cellular processes such as proliferation, differentiation, apoptosis and development.

snoRNAs are components of small nucleolar ribonucleoproteins (snoRNPs), which are responsible for the sequence-specific methylation and pseudouridylation of rRNA that takes place in the nucleolus. snoRNAs direct the assembled snoRNP complexes to a specific target (23).

Short ncRNAs have evolved following different rules than protein-coding genes. While the evolutionary pressure tends to maintain the translated sequence in protein-coding genes, in ncRNAs the pressure is in maintaining their secondary structure instead (32). Different mechanisms drive the expansion of these genes. In the case of snoRNAs, retroposition has been described as the major evolutionary force in the platypus and human genomes (33, 34) while intragenic duplication seems to be the main source of novel snoRNAs in chickens (35). Similarly miRNAs tend to evolve by intragenic duplication, followed by frequent losses soon after their formation (36). There are also significant differences in X-linked miRNAs, characterized by recent expansions by tandem duplications and rapid divergence (36).

Phylogenetic trees are commonly used to describe the evolution of individual genes; they play a fundamental part in gene and genome annotation (37–40). For example, phylogenetic trees are central for establishing reliable orthology and paralogy predictions (41), for elucidating the history and function of genes and for detecting relevant evolutionary events. Recent developments of faster algorithms and automated analysis methods for phylogenetic inference have enabled the computation of large sets of multiple sequence alignments and phylogenetic trees. These advances make it feasible to reconstruct the evolutionary history of all genes encoded in a given genome (42) or set of genomes (43–45). However, this analysis has generally been restricted to the protein-coding fraction of the genomes under study.

Genome-wide ncRNA orthology predictions have used synteny-based approaches in the past, which have been successful in many cases due to the tendency for short ncRNAs to maintain their intronic locations (46). The use of phylogenetic trees rather than synteny for ncRNAs orthology analysis requires special considerations because ncRNAs are often poorly conserved at the nucleotide level and homology is usually detected using secondary structure information with dedicated tools such as Infernal (47) and/or specialised ncRNA databases including Rfam (48) and mirBase (49). These databases provide sophisticated family descriptions to help in the identification, alignment and analysis of ncRNAs. For example, in Rfam ncRNA families are based on manually curated ‘seed alignments' expressed as covariance models (CMs), which provide a probabilistic description of both the secondary structure and the primary consensus sequence of an RNA (50). CMs are a natural extension of the profile Hidden Markov Models (pHMMs) that have been successfully applied to protein classification (51); CMs are reviewed in depth by Gardner (52). pHMMs are generated from seed alignments of representative members of a family of homologous sequences. Each column in the seed alignment is reduced to a vector of frequencies (probabilities) for each possible residue. The probabilities for each residue *x_i_* corresponding to a given sequence *X* are multiplied together to calculate the likelihood that the same processes that produced the seed alignment would have produced *X.* These probabilities are then used to find the best matches when aligning sequences to the model. pHMM concepts are applied to ncRNA sequences by explicitly adding information about RNA secondary structure to model constraints in RNAs, such as those arising from nucleotide pairing. The core models of existing CMs can be extended by aligning novel sequences with Infernal (47).

Compensatory substitutions in both nucleotides of paired regions of RNA helices conserve the molecule's structure and thus require CMs to accurately align ncRNAs. This pairing also has implications in the tree reconstruction of ncRNA alignments because most phylogenetic models assume that each site in a sequence evolves independently of the others: an assumption not valid for ncRNA genes. Current tree reconstruction methods are based on the probability of nucleotide replacements over evolutionary time. For this reason, neglecting the selection mechanisms which act for the maintenance of RNA stem-loops in RNA substitution models can strongly affect the estimation of likelihood of the plausible evolutionary scenarios in competition. To properly deal with this situation, RNA-specific substitution models have been developed. Like those developed for DNA, RNA substitution models are Markovian but they consider pairs of nucleotides as their elementary states rather than single sites (53). Models with 16 states can account for the 16 possible pairs that can be formed with four bases. Simpler models based on the 16-state models either discard mismatch pairs or lump them into a single state to create 6-state and 7-state Markov models, respectively. As with DNA substitution models, the best-fit model depends on the sequences being analysed.

Here, we present a new automated analysis method adapted to the special characteristics of ncRNAs, based on the Ensembl GeneTree pipeline (40). Ensembl's GeneTrees include multiple alignments and homology (orthology/paralogy) relationships for >50 eukaryotic genomes. The new method automatically identifies orthologues and paralogues in ncRNA gene families from phylogenetic trees. These are reconstructed by combining trees inferred using various secondary structure models from alignments of the RNA sequences and trees inferred using DNA-substitution models from alignments of the genomic loci. The former trees address the problem of compensatory mutations in the ncRNAs while the latter trees leverage information from the genomic context. By clustering, multiple alignment, phylogenetic tree inference and homology analysis of all ncRNA gene families present in the genomes included in Ensembl, we provide a consistent and comprehensive phylogenetic analysis of the ncRNA content of vertebrate genomes.

## Results and discussion

**Figure 1.**
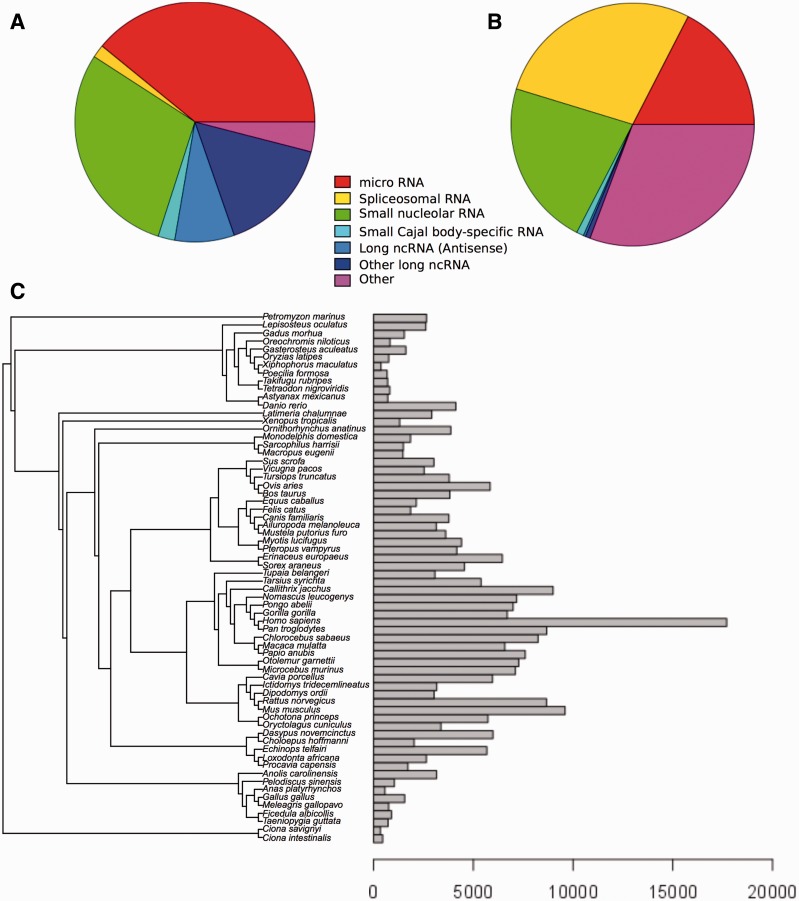
Distribution of Ensembl ncRNA genes in the Rfam database. (**A**) Distribution of Ensembl ncRNA gene families present in Rfam by family type. (**B**) Distribution of Ensembl ncRNA genes present in Rfam by family type. (**C**) Distribution of ncRNA genes by species.

Our robust and efficient method for ncRNA tree generation relies on the families described in Rfam. As of Ensembl release 82 (September 2015; http://e82.ensembl.org) there are 280 479 ncRNA genes annotated in all Ensembl species (see Methods), which correspond to 865 distinct Rfam families. Of the 2208 ncRNA families in Rfam (database version 11), only 768 contain at least 2 genes, accounting for a total of 119 130 Ensembl genes across all species; the remaining families mostly relate to non-vertebrate genes only or to genes in poorly characterized genomes that are filtered subsequently. Most families represent miRNA and snoRNA (Figure 1A), and the number of Ensembl genes belonging to each gene family varies substantially: some of the gene families have only a few genes, while others have thousands, with spliceosomal RNAs being the most abundant type of RNA in the trees we have generated (Figure 1B). For example, U6 and 5S_rRNA, classified as ‘Other’, contain 20 432 and 18 905 genes, respectively.

The distribution of annotated ncRNA genes per species also varies significantly (Figure 1C). Species such as sea squirts (*Ciona intestinalis* and *Ciona savignyi*) and platyfish (*Xiphophorus maculatus*) have very few annotated ncRNA genes, while primates generally have large numbers of annotated ncRNA genes. There are several factors that may explain this variability. First, some assemblies are more fragmented than others, which affects the quality of the annotation. Second, the ncRNA annotation process relies on comparative techniques (see Methods) meaning that species with a closely related genome having high-quality annotation will also benefit from this high-quality annotation. This effect explains the large number of ncRNAs that are annotated in primates, where ncRNA genes across the clade are inferred from the higher quality human ncRNA annotation. Other model species such as mouse (*Mus musculus*), rat (*Rattus norvegicus*) and zebrafish (*Danio rerio*) also have an increased set of ncRNAs compared to their phylogenetic neighbours.

Genomes with less contiguous assemblies (e.g. low-coverage genomes) typically contain an abundance of apparently duplicated regions because of assembly errors. This can result in an excess of spuriously annotated ncRNA genes. In these genomes, we filter ncRNA annotations based on genome-wide alignments in order to minimise the impact of assembly quality on the annotation of ncRNA. This is done by exploiting synteny using multiple alignments (see Methods): for Ensembl release 82, this resulted in the elimination of 83 851 genes across all species. A similar synteny-based strategy has been successfully used to estimate the origin of the human miRNA set (54) and in the future could potentially also be used to detect unannotated ncRNA genes.

**Figure 2.**
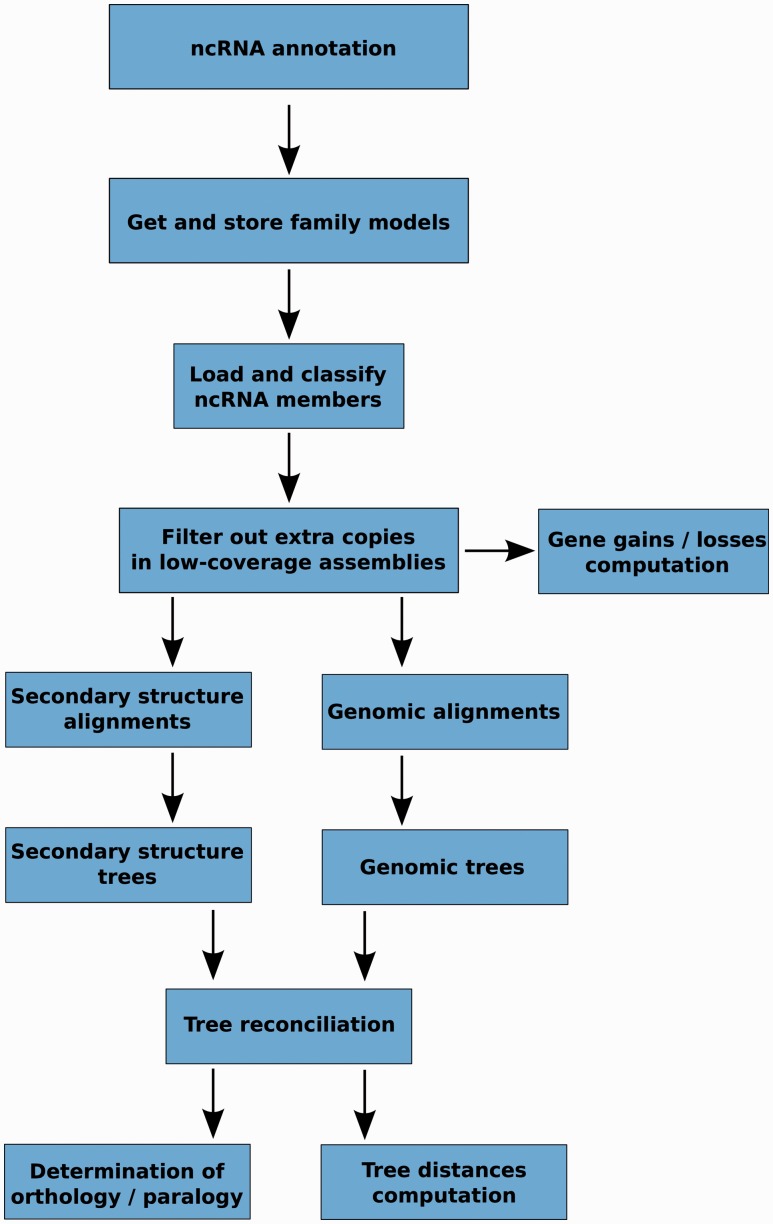
Schematic representation of the main steps in the ncRNA tree analysis pipeline.

The main steps of the pipeline are presented in Figure 2 and detailed information of each step is given in the Methods section. Briefly, these steps involve the identification and classification of all ncRNA genes based on the Rfam annotation, the alignment of the sequences within a gene family, the generation of several trees for each family, the merging of these intermediate trees in the light of the species tree and finally, the inference of orthology and paralogy relationships based on the final trees. One of the unique characteristics of our approach is the use of alternative alignment methods: we use Infernal to build alignments based on the secondary structure of the ncRNAs and PRANK to align the primary sequences, including the flanking regions.

The analysis is fully automated using the eHive system (55), which can process huge numbers of small jobs and run autonomously with minimal manual intervention. We have maximised parallelisation to take full advantage of highly distributed systems. For example, in the alignment and tree building phases, all gene families are processed in parallel; for each family, the different sets of alignments and trees are also computed in parallel. To improve performance we have also incorporated the latest developments in the eHive system, which includes semaphores to handle individual job dependencies.

### Gene family variability, supertrees and fast-trees

When aligning ncRNA genes, the secondary structure of the sequences has to be taken into account. We use Infernal to align all the ncRNA genes in a gene family (i.e. Rfam family). Based on the Infernal alignment, we build several ML trees using the standard nucleotide substitution model GTR-G and different 6-state (S6A, S6B, S6C, S6D, S6E); 7-state (S7A, S7B, S7C, S7D, S7E, S7F) and 16-state (S16, S16A, S16B) RNA base-paired substitution models (see Methods).

**Figure 3.**
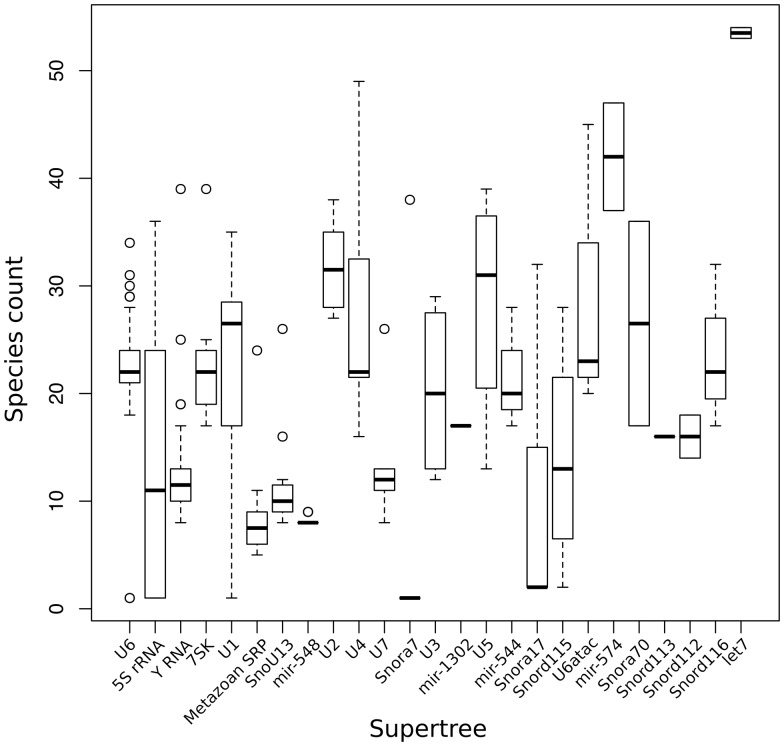
Distribution of number of species in the different sub-trees after splitting the super-trees.

As noted above, the size of the ncRNA gene families predicted in Ensembl varies substantially, both within and across genomes. In order to cope with this variability and still produce a reliable and efficient analysis, we split the gene families recursively into smaller groups that have up to 400 genes. For each of these groups, we infer an independent sub-tree. We build a ‘supertree' to re-connect all the subtrees belonging to the same gene family. This definition of supertree is slightly different from the one used for species tree reconstruction (56). The initial split of large families is based on a fast NJ tree build using quicktree (57) and the alignment of all genes in the gene family. The generation of this intermediate NJ tree is essential to minimise the separation of orthologous genes in the inferred subtrees. Figure 3 displays the number of species represented in the component subtrees that make up each of the 25 supertrees in Ensembl release 82. In general, the distributions are narrow showing that genes from different species are evenly distributed across the subtrees. Deviations can be explained by missing annotations in low-coverage genomes and lineage-specific expansions. When a gene family includes >150 kb of sequence in total, for practical reasons we use faster alternatives to the standard multiple aligners and tree building software to infer the trees (see Methods).

### Genomic alignments and trees

In addition to secondary structure-based trees we also include trees based on the primary sequence of the ncRNA genes. In this case, the ncRNAs are extended to include the genomic flanks of the gene prior to being aligned with PRANK (58, 59). PRANK is a phylogenetically aware multiple sequence aligner that relies heavily on the phylogenetic tree of the sequences being aligned. PRANK can produce its own guide tree using an NJ algorithm and evolutionary distances estimated from fast pairwise alignments. This guide tree can also be precomputed and provided directly to PRANK during program invocation.

**Figure 4.**
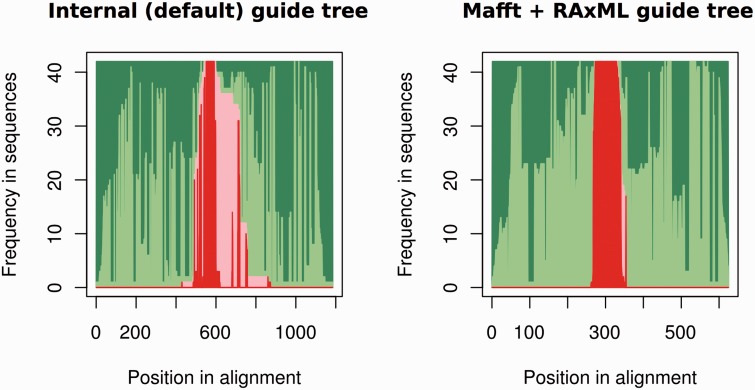
Summary of the PRANK alignment for the mir-652 gene family (17 genes) using either PRANK (default internal tree) or MAFFT + RAxML to build the guide tree. For each position in the alignment (x axis), we represent the fraction of gaps in flanking regions (dark green), aligned flanking sequence (light green), gaps in the ncRNA regions (light red) and aligned ncRNA regions (dark red). The figure shows, using MAFFT + RAxML to produce the guide tree, how we obtain an alignment where the ncRNA and the flanking regions are well segregated.

One way to assess the accuracy of the genomic alignments resulting from using either a pre-computed or self-generated guide tree is to check for the cross-alignment between the ncRNA sequences and their flanking regions. Ideally, all ncRNA genes would be properly ‘stacked’ in the alignments. Figure 4 shows an overview of the alignment for mir-652 both when allowing PRANK to build the guide tree and also when build the tree using external tools. The improved alignment when an ML tree is built with RAxML using the GTR-G model and provided to PRANK is evident by the reduced overlap between the ncRNAs and their flanking sequences. The alignments built using external trees are also shorter and more compact than the ones using the internal PRANK tree (Figure 4). Based on the PRANK alignments, we create one NJ and one ML tree (ga_nj and ga_ml respectively).

### Tree merging and reconciliation

**Figure 5.**
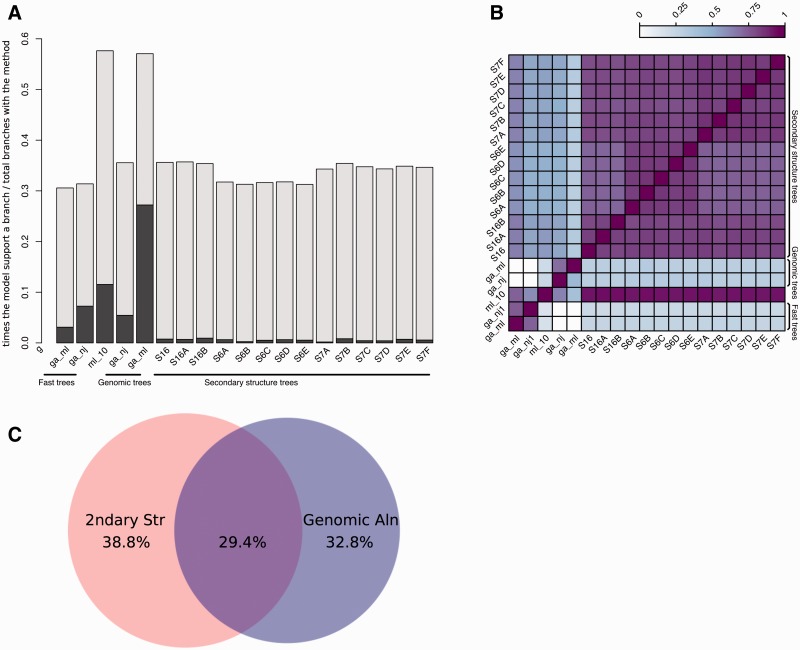
Analysis of tree reconciliation. (**A**) Intermediate tree support for each branch in the final tree. For each final branch in the final gene trees, the number of times a given intermediate tree supports a branch is calculated and divided by the total times that tree appears. The dark regions of each bar indicate the fraction of times the branch is supported only by that tree. (**B**) Heatmap representing the overlap between model support. The support for each model in all final branches in the final trees is divided by the union of models supporting them, i.e. when two models support the same final branches, this ratio is 1 and when no overlap is found, this ratio is 0. (**C**) Venn diagram showing the overlap between branches supported by trees based on secondary structure or genomic sequences. Fast trees are included in the corresponding category.

Up to 17 trees are built during the previous steps: 15 based on the secondary structure alignment and an additional two based on the genomic alignment. The merged trees are then reconciled with the species tree (see Methods), with the result that one or more input trees support each branch of the final tree. We measure the contribution of the different input trees by tracing which of them support a given branch in the final tree. This way we can study which models rarely support branches in the final tree and which ones tend to support the same branches. Figure 5A shows how often the intermediate trees support branches in the final trees. All models support some branches uniquely (Figure 5A; darker bars) although some support very few. For example, in the case of S6B and S7A the small number of uniquely supported branches is due to the similarity between its topology and that of the other trees, making these models less likely to contain branches not supported by other secondary structure trees.

In order to look for agreement between models, we calculated the overlap between each pair of models in the final trees. We define overlap as the number of branches supported by one of the models divided by the number of branches supported by other models. As summarised in Figure 5B, we observe that all secondary structure-based trees tend to have similar topography.

Figure 5A also shows that ML genomic trees (ga_ml) support the largest number of branches in the final trees. Interestingly, in half of these cases, it is the unique intermediate tree supporting the branch. Further analysis revealed that most of the final trees had at least one branch supported specifically by the ga_ml trees. Figure 5C shows the fraction of branches supported by secondary structure models only, by genomic alignments only, or both. We observe that most of the ncRNA trees that have branches better supported by genomic trees are intronic ncRNAs where the upstream and/or downstream sequences harbour coding sequence (data not shown). The genomic alignments based on these coding flanking regions give a powerful source of information in the phylogenetic reconstruction of these trees. All these data support the idea that both the genomic and secondary structure-based intermediate trees are contributing to improve the final trees.

**Figure 6.**
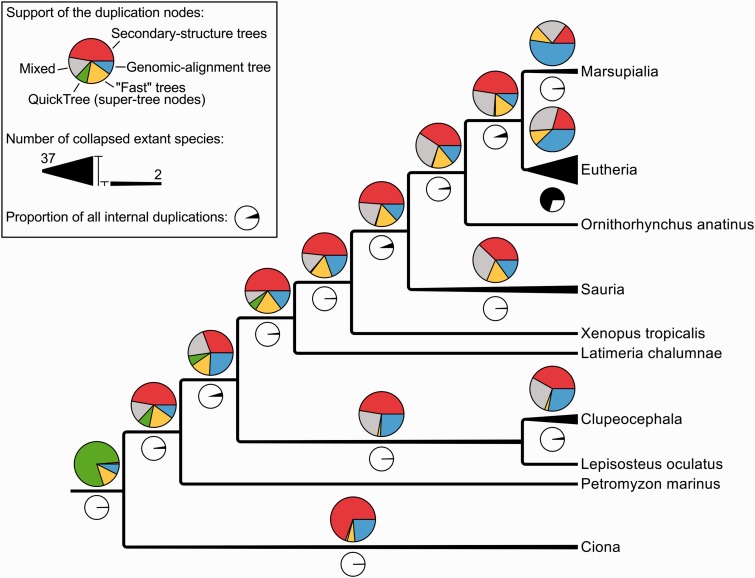
Simplified species-tree showing the support of all the internal duplications (coloured pie charts) and their numbers (black and white pie charts). ‘Mixed’ signifies that the duplication is supported by multiple kinds of intermediate trees, as opposite to the other labels such as ‘Secondary-structure trees’ which indicate that a duplication has been identified by a single kind of intermediate trees.

A closer look at the taxonomic annotation of duplications reveals that those branches are mostly (about 70%) Eutherian or more recent duplications (Figure 6; black pie-charts). About 40% of the Eutherian duplications are supported only by genomic alignments (blue section of the pie charts) and only 20% by secondary-structure alignments (red section of the pie charts). This contrasts with all the other nodes of the tree (except Marsupiala), which show the opposite trend. This suggests that Eutherian and Marsupialian duplications are better resolved using genomic alignments, likely because the variability in the flanking regions provides more information for resolving these duplications.

**Figure 7.**
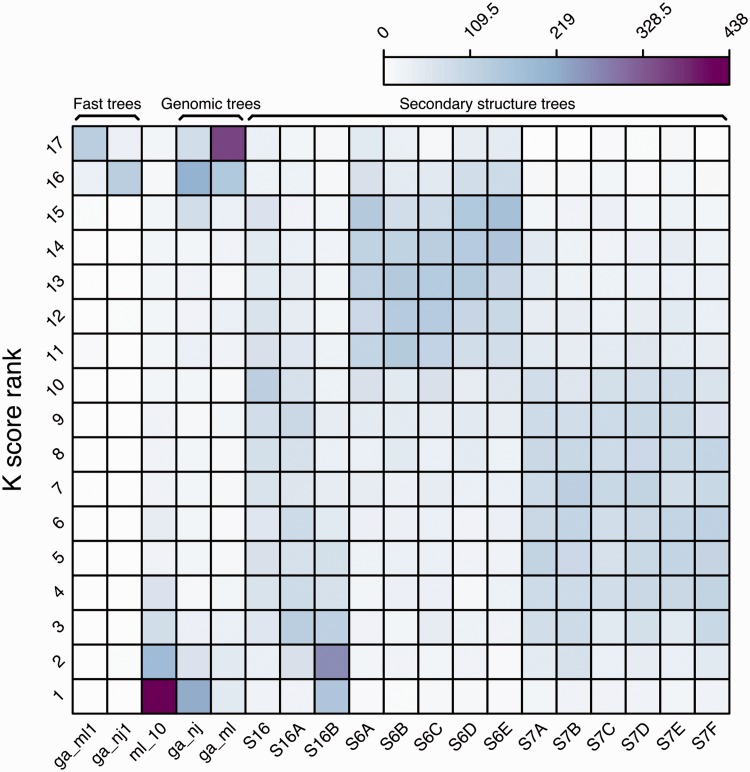
Ranking frequency of the different intermediate trees compared with the merged final tree based on their K tree scores.

We further analysed all the intermediate trees for each reconciled tree using K tree scores (60) (see Methods). The K tree score measures the overall differences in the relative branch length and topology of two phylogenetic trees by scaling one of the trees to have a global divergence as similar as possible to the other tree and calculating the minimum branch length distance between both. For each ncRNA family, all the intermediate trees were compared to the final merged tree and ranked using their K tree scores (Figure 7). The ml_10 trees are based on the secondary structure alignment, using the GTR-G model (see Methods) and are frequently the intermediate trees most similar to the final reconciled tree. In general, the intermediate trees based on 6-state models are ranked lower than the trees based on 7-state models.

Finally, the ML-based genomic tree (ga_ml) is the tree most frequently ranked in the last position (#17). Since Figure 5 shows that it is also the tree supporting more branches in the final trees, both uniquely or with other intermediate trees, we conclude that only some of the branches of these trees are contributing to the final tree (mostly Eutherian duplications) but, on the whole, the topology of the tree is not as similar to the final tree as are the other modelled phylogenies.

### Duplication confidence scores

**Figure 8.**
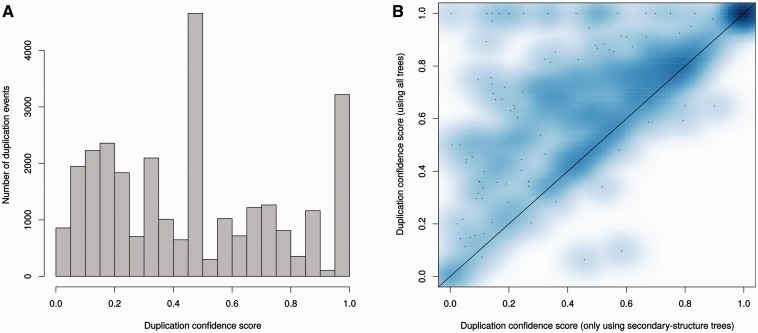
Analysis of duplication confidence scores in the resulting trees. (**A**) Distribution of confidence scores for non-species specific duplications determined by the ncRNA analysis pipeline including secondary structure trees, genomic-based trees and fast trees in Ensembl release 82. (**B**) Improvement of confidence scores for all duplications when genomic based intermediate trees are added to secondary structure-based trees in the merging step. Each data point in the heat map represents the average scores for a family.

It is expected that most duplications will leave the resulting duplicated genes present in subsequent lineages. During the reconciliation step it is therefore possible to detect poor tree topologies by searching for cases of predicted duplication events following extensive loss in the daughter lineages. The duplication confidence score (DCS) is defined as the fraction of species in which the duplication is detected (40). A low DCS identifies a poorly supported duplication event; a DCS equal to or near one is an indication of more parsimonious gene trees. Figure 8A shows the DCS of the ncRNA trees in Ensembl release 82, where we omit the species-specific duplications as their DCS is one by definition. A DCS of zero can happen when the gene and the species trees disagree. In order to limit the detrimental effect of the annotation bias among species, we simplify the species tree to reduce the number of potential contradictions in the tree (see Methods). As a result, a very small proportion of DCS have a value equal to zero.

As expected, genes are less conserved as the evolutionary distance increases, complicating the tree building process and increasing the proportion of dubious nodes in the older nodes. However, the proportion of duplication nodes with a confidence score of one is greater than that observed for protein trees (Supplementary Figure 1) using the Ensembl GeneTree pipeline (40), demonstrating the high quality of the predicted duplications for ncRNA trees. Detailed statistics are available on the Ensembl website at (http://e82.ensembl.org/info/docs/compara/nc_tree_stats.html).

We used the same criteria to assess the impact of adding genomic alignments. We took all the families with intermediate trees based on secondary structure and genomic alignments and computed the final trees using either only secondary structure or both alignments. We then compared the DCS of the two sets of final trees: the DCS were enhanced with the addition of genomic trees (Figure 8B), which is consistent with our previous observation of the genomic trees being especially useful to resolve Eutherian duplications (Figure 6).

### Determination of orthology/paralogy

The reconciled tree is used to infer orthology and paralogy relationships by comparing every gene with every other gene in the tree. Paralogous genes are related by a duplication event and, of all possible paralogues, we mainly identify *within-species paralogues*, which are a pair of genes from the same species. Orthologous genes are related by a speciation event and we annotate them in the following classes. Specifically, *one-to-one orthologues* are orthologues found as a single copy in each species. *One-to-many orthologues* are orthologues that have been duplicated in one of the two lineages since the speciation event and *many-to-many orthologues* are orthologues that have been duplicated in both lineages. We also annotate as orthologues pairs of genes for which there is no better match in those two species and that are related by an ill-supported duplication node (duplication confidence score below 25%). They are also classified into *one-to-one*, *one-to-many* and *many-to-many* and overall form the final set of orthologues.

A summary of the ncRNA orthology relationship between human and other selected species for Ensembl release 82 is shown in Table 1. As expected, we find mostly one-to-one orthologues between primates. In contrast, we can only find 34 one-to-one orthologues between the human and lamprey genomes. Out of the 881 one-to-one human-mouse gene pairs, 37% are spliceosomal ncRNAs, 23% are small nucleolar ncRNAs and 13% are lnRNAs, leaving out 23% of other unclassified RNAs. 1192 human genes are related by more complex orthology relationships with mouse. As mentioned earlier, the quality of ncRNA annotation in the different species has a large impact on the number of orthologues and paralogues that can be found. In addition, we predict 6811 ncRNA within-species paralogues in human, 6897 ncRNA paralogues in chimp, 8178 in marmoset, 3641 in mouse, 438 in zebra finch and 2637 in zebrafish.

**Table 1.** . Number of one-to-one, one-to-many, many-to-many determined in the ncRNA pipeline for all the human ncRNAs

Human - VS	1-to-1	1-to-many	many-to-many
Chimp	5497	288	132
Marmoset	3293	821	235
Mouse	881	914	278
Zebra finch	202	468	141
Zebrafish	133	355	341

To assess the quality of our orthology predictions, we looked for syntenic pairs of protein orthologues in the vicinity of each ncRNA orthologue found between human and selected species. For each pair of species, we considered all pairs of predicted ncRNA orthologues. If the ncRNAs were located inside other protein-coding genes (i.e. in introns) we investigated whether both protein genes were also orthologues as predicted in our protein gene trees (40). In the cases of other pairs of ncRNA orthologues, we looked for pairs of protein orthologues in the vicinity of 5 kb both upstream and downstream of the ncRNA genes with the same orthology type (‘one-to-one’ or ‘one- to-many’ orthologues). The results (Table 2), show that most of the human-chimpanzee and human-marmoset ncRNA orthologues are located in or near to protein orthologues with the same orthology relationship. These results show coherence between the orthology predictions for protein-coding and ncRNA genes.

**Table 2.** . Number of ncRNA pair of orthologs in or near protein orthologs with the same orthology relationship in the selected pairs of species

Orthologs	% Syntenic protein orthologues (intronic)	% Syntenic protein orthologues (5 kb)
Human–Chimp	1870/1948 (96.0%)	387/430 (90.0%)
Human–Marmoset	956/1256 (76.1%)	191/313 (61.0%)
Human–Mouse	205/682 (30.1%)	83/219 (37.9%)
Human–Zebra finch	121/233 (51.9%)	30/89 (33.7%)
Human–Chicken	175/302 (58.0%)	46/112 (41.1%)
Human–Zebrafish	114/434 (26.3%)	15/85 (17.6%)

### Determination of gene gains and losses

**Figure 9.**
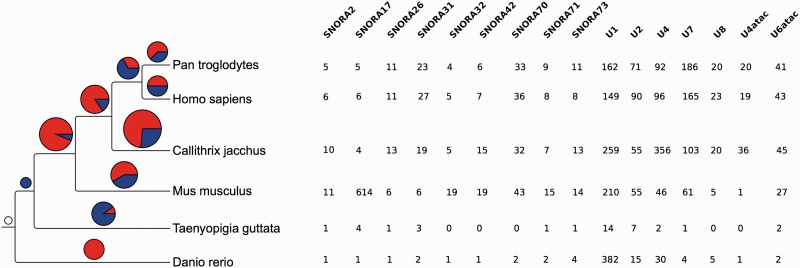
Gene family expansions and contractions. The tree on the left shows the species used in the gene family evolution of ncRNA trees. The pie charts show the number of gene families expanded (red) and contracted (blue) in each node of the tree. The size of the pie chart is proportional to the number of families that have expanded or contracted. The table on the right shows the families expanded in the mammal lineage. The numbers indicate the number of genes in each extant species.

In addition to the inferred phylogenetic trees, we also estimated the rates of apparent gene gain and loss in each family. To minimise the effect of annotation bias, we restrict this analysis to the human, chimp, mouse, marmoset, zebra finch and zebrafish genomes based on the quality of their annotation and their phylogenetic distribution. For these estimations we used CAFE (61, 62), which models gene family evolution as a stochastic birth-and-death process where genes are gained and lost independently along each branch of a phylogenetic tree. We only consider genes that can be detected at the root of the tree (i.e. the most recent common ancestor of the gene in the species tree is the root of the tree). In CAFE, the λ parameter describes the rate of change as the probability that a gene family either expands or contracts (via gene gain and loss) per gene per million years. CAFE allows for the λ parameter to be estimated separately for independent branches of the phylogenetic tree. When analysing gene gains and losses in each branch we observed an apparent expansion of gene families in all the mammalian branches (Figure 9). While annotation biases can affect these results, H/ACA small nucleolar RNAs and spliceosomal RNAs seemed to be expanded in mammals relative to the other species studied. The CAFE analysis provides a useful summary of annotated genes per species.

Having organised all the genes in families across all Ensembl species, it is possible to search for lineage-specific genes. For instance, we count 15 miRNAs present in all the primates but absent in any other species considered. Most (11/15) of these miRNAs are located in introns of other genes, which is consistent with previous reports of the predominance of human miRNA loci located within intronic regions (46, 63) and the observation that this positioning has been conserved during mammalian evolution (46). Table 3 lists the possible target genes of these miRNAs using two independent databases: miRNAmap2.0 (64) and miRNA (www.miRNA.org). Incidentally, the list of target genes contains many transcriptional regulators including several zinc finger proteins such as PRDM2, ZNRF2 and ZNF512 as well as other DNA binding proteins including TARDBP. However, we found the target genes predicted using both databases were strikingly different, with no miRNA gene having the same target predicted by both databases for the same gene.

**Table 3.** Primates-specific microRNAs

Gene	Copies in Human	Target Genes (miRNAmap)	Description of target genes (miRNAmap)	Target Genes (microRNA)	Description of target genes (microRNA)	Location of miRNA
mir-550	3	PRDM2	PR domain zinc finger protein 2	LGI1	leucine-rich, glioma inactivated 1 (LGI1)	+ Inside intron of gene ZNRF2 (zinc and ring finger 2)
						+ Inside intron of AVL9 homolog from S.cerevisiae
		FUSIP1	FUS-interacting serine-arginine-rich protein 1	SHISA2	Shisa homolog 2 (Xenopus laevis)	
				POU2F1	POU class 2 homeobox 1	
mir-556	1	KIF1B	Kinesin-like protein KIF1B (Klp)			+ Inside intron of gene NOS1AP (nitric oxide synthase 1 (neuronal) adaptor protein)
		TARDBP	TAR DNA-binding protein 43 (TDP-43)			
		LYPLA2	Acyl-protein thioesterase 2			
mir-573	1	CPSF3L	Integrator complex subunit 11	LARP1B	La ribonucleoprotein domain family, member 1B	+Intergenic
				C10orf118	Chromosome 10 open reading frame 118	
				SLC25A26	Solute carrier family 25, member 26	
				CCDC62	coiled-coil domain containing 62	
				ST6GALNAC3	ST6 (alpha-N-acetyl-neuraminyl-2,3-beta-galactosyl-1, 3)-N-acetylgalactosaminide alpha-2,6 sialyltransferase 3	
mir-580	1	PARK7	Protein DJ-1 (Oncogene DJ1) (Parkinson disease protein 7)	ZBTB1	Zinc finger and BTB domain containing 1	+Inside intron of gene LMBR1D2 (LMBR1 domain containing 2)
		ALPL	Alkaline phosphatase, tissue-nonspecific isozyme precursor	EPB41L2	Erythrocyte membrane protein band 4.1-like 2	
				MRPL42	Mitochondrial ribosomal protein L42	
				PYROXD1	Pyridine nucleotide-disulphide oxidoreductase domain 1	
mir-581	1	FRAP1	FKBP12-rapamycin complex-associated protein (FK506-binding protein 12- rapamycin complex-associated protein 1) (Rapamycin target protein) (RAPT1) (Mammalian target of rapamycin) (mTOR)	THAP1	THAP domain containing, apoptosis associated protein 1	+Inside intron of gene ARL15 (ADP-ribosylation factor-like 15)
				RBM6	RNA binding motif protein 6	
				MYO10	myosin X	
				CCDC66	Coiled-coil domain containing 66	
mir-583	1	SPSB1	SPRY domain-containing SOCS box protein 1 (SSB-1)	CCDC141	Coiled-coil domain containing 141	+Intergenic
		SPEN	Msx2-interacting protein (SPEN homolog) (SMART/HDAC1-associated repressor protein).	ZNF512	zinc finger protein 512	
		CDC42	Cell division control protein 42 homolog precursor (G25K GTP-binding protein).	WNK3	WNK lysine deficient protein kinase 3	
mir-586	1	ZUBR1	retinoblastoma-associated factor 600	FAM164A/ZC2HC1A	zinc finger, C2HC-type containing 1A	+Inside intron of gene SUPT3H (suppressor of Ty 3 homolog (S. cerevisiae))
		FAM76A	Protein FAM76A	MMP13	Matrix metallopeptidase 13 (collagenase 3)	
		STX12	Syntaxin-12	ITPR1	Inositol 1,4,5-trisphosphate receptor, type 1	
mir-597	1	CCNL2	Cyclin-L2 (Paneth cell-enhanced expression protein)	SMARCE1	SWI/SNF related, matrix associated, actin dependent regulator of chromatin, subfamily e, member 1	+Inside intron of gene TNKS1 (tankyrase, TRF1-interacting ankyrin-related ADP-ribose polymerase)
		UBE4B	Ubiquitin conjugation factor E4 B (Ubiquitin fusion degradation protein 2) (Homozygously deleted in neuroblastoma 1)			
mir-601	1	PPP1R8	Nuclear inhibitor of protein phosphatase 1 (NIPP-1) (Protein phosphatase 1 regulatory inhibitor subunit 8)	PMCHL2	pro-melanin-concentrating hormone-like 2, pseudogene (ncRNA)	+Inside intron of gene DENND1A (DENN/MADD domain containing 1A)
		ZBTB8	Zinc finger and BTB domain-containing protein 8			
		FHL3	Four and a half LIM domains protein 3 (FHL-3) (Skeletal muscle LIM- protein 2) (SLIM 2)			
mir-605	1	TARDBP	TAR DNA-binding protein 43 (TDP-43)	MTRR	5-methyltetrahydrofolate-homocysteine methyltransferase reductase	+Inside intron of gene PRKG1 (protein kinase, cGMP-dependent, type I)
		RPA2	Replication protein A 32 kDa subunit (RP-A) (RF-A) (Replication factor-A protein 2) (p32) (p34)	SERTAD4	SERTA domain containing 4	
		TAF12	Transcription initiation factor TFIID subunit 12 (Transcription initiation factor TFIID 20/15 kDa subunits) (TAFII-20/TAFII-15) (TAFII20/TAFII15)	LUC7L3	LUC7-like 3 (S. cerevisiae)	
mir-624	1	PANK4	Pantothenate kinase 4	NBEA	Neurobeachin	+Inside intron of gene STRN3 (striatin, calmodulin binding protein 3)
		TARDBP	TAR DNA-binding protein 43 (TDP-43)	SS18	Synovial sarcoma translocation, chromosome 18	
		ARID1A	AT-rich interactive domain-containing protein 1A (ARID domain-containing protein 1A) (SWI/SNF-related, matrix-associated, actin-dependent regulator of chromatin subfamily F member 1) (SWI-SNF complex protein p270) (B120) (SWI-like protein)	PPP6R3	Protein phosphatase 6, regulatory subunit 3	
mir-640	1	KIF1B	Kinesin-like protein KIF1B (Klp)	ZDHHC17	Zinc finger, DHHC-type containing 17	+Inside intron of gene GATAD2A (GATA zinc finger domain containing 2A)
		VPS13D	Vacuolar protein sorting-associated protein 13D	SLC30A5	Solute carrier family 30 (zinc transporter), member 5	
				KCTD18	Potassium channel tetramerisation domain containing 18	
mir-648	1	RERE	Arginine-glutamic acid dipeptide repeats protein (Atrophin-1-like protein) (Atrophin-1-related protein)	HBP1	HMG-box transcription factor 1	+Intergenic
		KIF1B	Kinesin-like protein KIF1B (Klp)	MOG	Myelin oligodendrocyte glycoprotein	
mir-651	1	RER1	Protein RER1	ITGB1	integrin, beta 1 (fibronectin receptor, beta polypeptide, antigen CD29 includes MDF2, MSK12)	+Intergenic
				SPDYE8P	Speedy homolog E8 (Xenopus laevis)	
				BMP2K	BMP2 inducible kinase	
mir-887	1			P2RY12	Purinergic receptor P2Y, G-protein coupled, 12	+Inside intron of gene FBXL7 (F-box and leucine-rich repeat protein 7)

The target genes predicted by miRNAmap2.0 and microRNA and their description are shown.

### Web display and access to data

**Figure 10.**
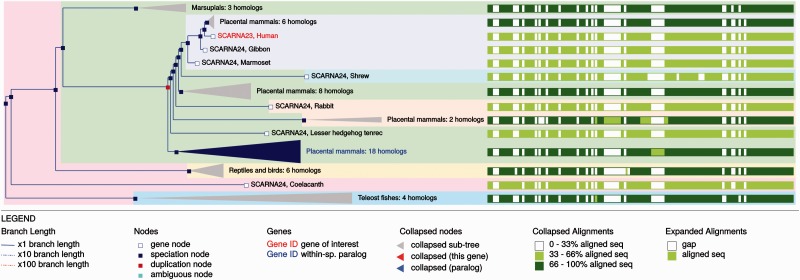
Example gene tree displayed in the Ensembl genome browser.

The ncRNA trees and homology information are updated with every Ensembl release and can be visualised through the Ensembl genome browser. The main entry point for the trees is the Gene Tree for each gene (e.g. http://e82.ensembl.org/Homo_sapiens/Gene/Compara_Tree?collapse= none;g= ENSG00000251869): this view displays the gene tree and highlights the query gene. Sub-trees can be expanded or contracted to better visualise different parts of the tree. Duplication nodes are coloured red, whereas speciation nodes are shown in blue. For example, in Figure 10 we show the tree for the SCARNA23 gene. The tree shows the duplication events leading to several copies of the gene in primates. The tree can be exported as pdf, svg, ps or png files. The orthology/paralogy information and pairwise and multiple alignments between different set of orthologues are also available in specialized views. We also provide a gene gain/loss tree view for the CAFE trees and results.

The ncRNA orthology and paralogy data described here can be downloaded directly from the Ensembl FTP site. For release 82, the directories are ftp://ftp.ensembl.org/pub/release-82/emf/ensembl-compara/homologies/ and ftp://ftp.ensembl.org/pub/release-82/xml/ensembl-comp ara/homol ogies/. Data are provided in four widely used formats: FASTA to retrieve the aligned sequences; PhyloXML (65) and Newick to retrieve the trees; and OrthoXML (66) to access either the orthology groups or the orthology pairs. Available file types also include Ensembl Multi Format (EMF), a format developed before PhyloXML and OrthoXML were available. The two FTP directories have informative README files describing their content. Programmatic access to the data is also provided through the Ensembl API (http://www.ensembl.org/info/docs/api/compara/).

## Conclusions

Over the past 10 years, it has become clear that ncRNAs play a key role in cellular processes. Several classes of ncRNAs, such as small interfering RNAs, miRNAs, Piwi-associated RNAs, small nucleolar RNAs and transcribed ultra-conserved regions are implicated in cancer, heart diseases, immune disorders and neurodegenerative and metabolic diseases. Numerous ncRNAs have been found to have a role in gene regulation and are consequently emerging as therapeutic targets.

The number of resources available for ncRNAs is very limited, especially when compared to those available for protein-coding genes. While there are efforts to improve the access to these data (67) and several outstanding resources including Rfam (48) and miROrtho (68) have been made available. However, these only provide family classification and multiple alignments. We are not aware of any other resource that includes phylogenetic trees for ncRNAs and describes orthology and paralogy relationships of a wide spectra of ncRNAs.

Resolving the phylogeny of short ncRNA genes is a difficult task. We have developed a comprehensive methodology to address this, which produces an automated set of phylogenetic gene trees and orthology/paralogy relationships between ncRNA genes in vertebrate genomes. Our approach combines up to 17 different trees for each family, and we have shown the usefulness of combining different RNA evolutionary models. While there are many similarities among the different models, there is not a single model that resolves satisfactorily all the trees. In addition, the inclusion of genomic alignments of the ncRNA and their flanking regions helps to improve the trees, specifically by resolving some difficult duplication events.

The analysis and annotation method presented here is being actively maintained as part of the Ensembl project. New features and improvements are being added continuously. For example, we recently added a new view on the Ensembl genome browser that displays the secondary-structure plots of each ncRNA, including sequence conservation from the multiple-sequence alignment. In terms of data generation, we are currently testing improvements in the determination of gene gains/losses using alternatives to CAFE such as BadiRate (69) or COUNT (70). We plan to use the alignments, trees and orthology predictions to further refine the annotation of these genes. This will involve assessing the quality of the gene models using the alignments and looking for missing genes by focusing on lineage-specific gene loss events. We also plan to develop an alternative version of the analysis pipeline that will support the addition of a new species to the existing set of trees and orthologs.

## Methods

### ncRNA annotation

Scanning the entire genome with all the Rfam CMs would be prohibitively CPU intensive. Thus, ncRNAs are predicted using an automatic pipeline involving the following steps. First, a combination of several sensitive BLAST searches are used to identify likely targets, then a CM search using Infernal and the Rfam models is used to measure the probability that the targets can fold into the required structures. This two-step procedure reduces the search space and therefore limits the computational requirements. Other ncRNAs are added as described below. The following non-coding RNA gene types are annotated: tRNA, mt-tRNA, rRNA, scRNA, snRNA, snoRNA, miRNA, misc_RNA and lincRNA, but scRNA, snRNA, snoRNA and miRNA are the only short ncRNA used to infer phylogenetic relationships (http://www.ensembl.org/info/docs/genebuild/ncrna.html). Starting from Rfam 11 also includes models for lncRNAs (71). The genes matching these models are also considered here.

MiRNAs are predicted by BLASTN of genomic sequence slices against miRBase (49) sequences. The BLAST hits are clustered and filtered by e-value and the aligned genomic sequence is then checked for possible secondary structure using RNAfold (72). If evidence is found that the genomic sequence could form a stable hairpin structure, the locus is used to create a miRNA gene model. Finally, the resulting miRNA predictions are mapped to Rfam entries. tRNAs in the mitochondrial genome are annotated using tRNAscan-SE (73). Human and mouse lincRNAs are annotated using cDNA alignments and chromatin-state map data from the Ensembl regulatory build following a similar strategy as described in Guttman et al (74). Briefly, regions of chromatin methylation (H3K4me3 and H3K36me3) outside known protein-coding loci are identified. Next, cDNAs which overlap with these regions are considered lincRNAs candidates. A final evaluation step investigates if each of these candidates has any protein-coding potential, rejecting candidates containing a substantial open reading frame (ORF) covering at least 35% of its length and PFAM/tigrfam protein domains.

### Filtering step

Less complete and fragmented assemblies may contain a large number of redundant ncRNA annotations. To avoid introducing these extra copies, we filter out additional copies in these assemblies using our 39-way EPO multiple alignments (44). When several copies of an ncRNA are predicted in a low-coverage genome, we use the genomic alignments to detect the one located in the locus with most sequence identity. Only this copy is kept and the remaining copies are discarded.

We require any family to have at least three genes to proceed with the phylogenetic inference and analysis.

### Secondary structure alignments

A secondary structure-based alignment for each family is performed using Infernal (version 1.1) (47) and the corresponding CM. Initially, all the ncRNAs annotated in a family are aligned to its family model using *cmalign* with -mxsize = 4000 and default values for all other options. Next, the alignment is used to refine the model using cmbuild with the refine option. Finally, the sequences are realigned and a new model is created based on the new alignment. This process is repeated until convergence, i.e. when two successive iterations yield nearly identical alignments.

### ncRNA trees based on secondary structure alignments

Based on the Infernal alignments we build several ML trees using different models. The alignments and the structure files obtained with Infernal are then used to build several phylogenetic trees. Initially, one ML tree is performed with RAxML version 7.2.8-ALPHA (HPC-SSE3) (75) using a generic bootstrap value of 10 and the GTR-G model (ml_10). Additional phylogenetic trees are built using 6-state, 7-state and 16-state models from RAxML, adjusting the bootstrap value to take into account the time needed to build the first tree (with minimum and maximum values of 10 and 100 respectively) for resource optimisation. A thorough explanation of the differences between these models is available in the PHASE (76) software documentation (http://www.bioinf.man.ac.uk/resources/phase/).

### Genomic trees

In addition to trees based on secondary structure alignments, we build trees based on the genomic sequence of the ncRNAs. For these alignments we extend the nucleotide sequence of the gene by twice its length on both the 5' and 3' ends of the ncRNA. These alignments are especially relevant when the ncRNA sequence is not enough to resolve the phylogeny of the family. We use PRANK (58) to build these alignments. We provide PRANK with a guide tree built using MAFFT to pre-align the sequences and RAxML to estimate the tree. MAFFT is run with the auto option. We use the following options for RAxML: ‘-m GTRGAMMA -N 10’. PRANK is run using ‘-noxml -notree -once -f = Fasta’.

These genomic alignments are then used to build one NJ (ga_nj) and one ML (ga_ml) tree using TreeBeST (https://github.com/Ensembl/treebest).

### Fast trees

The number of ncRNA genes per model is extremely variable. 180 ncRNA gene families are too large (>150 kb of input sequence) to build genomic trees with the aforementioned strategy in a reasonable amount of time. For these families we build fast trees using FastTree2 (77) with options -nt -quiet -nopr and RaxML-Light (version 1.0.6) (78) in combination with Parsimonator v.1.0.2 (http://sco.h-its.org/exelixis/software.html), a lightweight and fast implementation for building starting trees for RAxML under parsimony (with options -p 12345). When built based on secondary structure alignments the trees are called ss_nj and ss_ml (using FastTree2 and RAxML-Light respectively); the trees based on genomic alignments are called ga_ml and ga_nj like their counterparts.

### Tree merging and reconciliation

For each family we reconcile the gene trees with the species tree using the *mmerge* function in TreeBeST (https://github.com/Ensembl/treebest). The species tree is a pruned version of the NCBI Taxonomy database (79), where only the species that are present in Ensembl are kept. In addition, we simplify the Eutheria, Sauria and Clupeocephala sub-trees by removing all internal nodes. This gives TreeBeST more flexibility when choosing a topology within these clades and avoid over-calling duplication nodes in those lineages. In the merging phase, all the input trees are rooted and the length of each branch is calculated with the *nj* mode using the -l option. This option was added in-house to allow us to track which input trees support which branch in the final tree. The mmerge algorithm of TreeBeST recursively divides a set of genes into two subsets given multiple reconciled gene trees as the input. mmerge starts from the whole set of genes and thus builds a binary gene tree. The algorithm is very efficient because it only has to consider partitions found in the input trees. It first favours speciation nodes over duplication nodes and then tries to minimise the number of gene loss events that would have been inferred in the two branches. Finally, it uses the bootstrap information to resolve the remaining ties.

In each step, the list of input trees supporting the final split is recorded. This information is used for analysing the consistency of the input trees.

TreeBeST also roots the gene trees. The approach is similar to the mmerge algorithm: it first finds the root that will minimise the total number of duplications and gene losses in the tree. Only the topology of the tree is used at this stage: sequence-related measures, such as the branch lengths or the actual alignment, are ignored. For more details on TreeBeST algorithms, see: http://lh3lh3.users.sourceforge.net/download/PhD-thesis-liheng-2006-English.pdf.

### Tree distances

For each family, the final tree is compared with its input trees using the program Ktreedist (60) with the -a option. This program calculates the minimum branch length distance between phylogenetic trees. This branch length is also called the “K tree score” and provides a measure of the distance in both topology and branch lengths between two trees. The input trees were re-rooted with TreeBeST using the *sdi* mode.

### Gene gain and losses

For these analyses we used version 2.2 of CAFE (61), which supports the estimation of independent rates along individual branches of the phylogenetic tree. We calculate the lambda for the tree in an iterative way. Firstly, it creates the newick-formatted ultrametric species tree where the branch lengths represent integer units of time. Secondly, a table containing the number of genes per gene family per species is created. This table only contains those gene families having at least one gene in the root of the species tree, i.e. the lowest common ancestor of the gene family is the root of the tree. Thirdly, CAFE is run using the option ‘lambda -s’. The output is then parsed. If the program fails to calculate a proper lambda (i.e. the log likelihood of the data for all families is not maximised), we filter out from the table the gene families with outlier values and a new lambda is calculated. This process is repeated until the lambda value is properly maximised. Finally we run CAFE, using this lambda value, with the original table containing all gene families that have at least one gene in the root node. The species tree used in this analysis is an Ensembl species tree where the multifurcated nodes are disambiguated and the branches annotated with their divergence times, in millions of years, using TimeTree (80). This process results in an ultrametric binary species tree.

## Supplementary data

Supplementary data are available at *Database* Online.

Supplementary Data

## References

[1] The ENCODE Project Consortium (2012) An integrated encyclopedia of DNA elements in the human genome. Nature, 489, 57–74. 22955616 10.1038/nature11247 PMC3439153

[2] Washietl S. Pedersen J.S. Korbel J.O (2007) Structured RNAs in the ENCODE selected regions of the human genome. Genome Res., 17, 852–864. 17568003 10.1101/gr.5650707 PMC1891344

[3] Kishore S. Stamm S. (2006) The snoRNA HBII-52 regulates alternative splicing of the serotonin receptor 2C. Science, 311, 230–232. 16357227 10.1126/science.1118265

[4] Billi A.C. Freeberg M.A. Kim J.K. (2012) piRNAs and siRNAs collaborate in Caenorhabditis elegans genome defense. Genome Biol., 13, 164 22818087 10.1186/gb-2012-13-7-164 PMC3491375

[5] Lee H.C. Gu W. Shirayama M (2012) C. elegans piRNAs mediate the genome-wide surveillance of germline transcripts. Cell, 150, 78–87. 22738724 10.1016/j.cell.2012.06.016 PMC3410639

[6] Simon M.D. Wang C.I. Kharchenko P.V (2011) The genomic binding sites of a noncoding RNA. Proc. Natl. Acad. Sci. USA, 108, 20497–20502. 22143764 10.1073/pnas.1113536108 PMC3251105

[7] Yang L. Lin C. Liu W (2011) ncRNA- and Pc2 methylation-dependent gene relocation between nuclear structures mediates gene activation programs. Cell, 147, 773–788. 22078878 10.1016/j.cell.2011.08.054 PMC3297197

[8] Chen K. Rajewsky N. (2007) The evolution of gene regulation by transcription factors and microRNAs. Nat. Rev. Genet., 8, 93–103. 17230196 10.1038/nrg1990

[9] Croce C.M. (2009) Causes and consequences of microRNA dysregulation in cancer. Nat. Rev. Genet., 10, 704–714. 19763153 10.1038/nrg2634 PMC3467096

[10] Nicoloso M.S. Spizzo R. Shimizu M (2009) MicroRNAs — the micro steering wheel of tumour metastases. Nat. Rev. Cancer, 9, 293–302. 19262572 10.1038/nrc2619

[11] Gehrke S. Imai Y. Sokol N (2010) Pathogenic LRRK2 negatively regulates microRNA-mediated translational repression. Nature, 466, 637–641. 20671708 10.1038/nature09191 PMC3049892

[12] Faghihi M.A. Modarresi F. Khalil A.M (2008) Expression of a noncoding RNA is elevated in Alzheimer’s disease and drives rapid feed-forward regulation of beta-secretase. Nat. Med., 14, 723–730. 18587408 10.1038/nm1784 PMC2826895

[13] Hébert S.S. Horré K. Nicolaï L (2008) Loss of microRNA cluster miR-29a/b-1 in sporadic Alzheimer’s disease correlates with increased BACE1/beta-secretase expression. Proc. Natl. Acad. Sci. USA, 105, 6415–6420. 18434550 10.1073/pnas.0710263105 PMC2359789

[14] Wang W.X. Rajeev B.W. Stromberg A.J (2008) The expression of microRNA miR-107 decreases early in Alzheimer’s disease and may accelerate disease progression through regulation of beta-site amyloid precursor protein-cleaving enzyme 1. J. Neurosci., 28, 1213–1223. 18234899 10.1523/JNEUROSCI.5065-07.2008 PMC2837363

[15] Yang B. Lin H. Xiao J (2007) The muscle-specific microRNA miR-1 regulates cardiac arrhythmogenic potential by targeting GJA1 and KCNJ2. Nat. Med., 13, 486–491. 17401374 10.1038/nm1569

[16] Zhao Y. Ransom J.F. Li A (2007) Dysregulation of cardiogenesis, cardiac conduction, and cell cycle in mice lacking miRNA-1-2. Cell, 129, 303–317. 17397913 10.1016/j.cell.2007.03.030

[17] Esteller M. (2011) Non-coding RNAs in human disease. Nat. Rev. Genet., 12, 861–874. 22094949 10.1038/nrg3074

[18] Brosius J. (2005) Waste not, want not–transcript excess in multicellular eukaryotes. Trends Genet., 21, 287–288. 15851065 10.1016/j.tig.2005.02.014

[19] Griffiths-Jones S. (2007) Annotating noncoding RNA genes. Annu. Rev. Genomics Hum. Genet., 8, 279–298. 17506659 10.1146/annurev.genom.8.080706.092419

[20] Sun Y. Aljawad O. Lei J (2012) Genome-scale NCRNA homology search using a Hamming distance-based filtration strategy. BMC Bioinformatics, 13 Suppl 3, S12 22536896 10.1186/1471-2105-13-S3-S12 PMC3311100

[21] Yandell M. Ence D. (2012) A beginner’s guide to eukaryotic genome annotation. Nat. Rev. Genet., 13, 329–342. 22510764 10.1038/nrg3174

[22] Carninci P. Kasukawa T. Katayama S (2005) The transcriptional landscape of the mammalian genome. Science, 309, 1559–1563. 16141072 10.1126/science.1112014

[23] Bachellerie J.P. Cavaillé J. Hüttenhofer A. (2002) The expanding snoRNA world. Biochimie, 84, 775–790. 12457565 10.1016/s0300-9084(02)01402-5

[24] Brennecke J. Aravin A.A. Stark A (2007) Discrete small RNA-generating loci as master regulators of transposon activity in Drosophila. Cell, 128, 1089–1103. 17346786 10.1016/j.cell.2007.01.043

[25] St Johnston D. (2005) Moving messages: the intracellular localization of mRNAs. Nat. Rev. Mol. Cell Biol., 6, 363–375. 15852043 10.1038/nrm1643

[26] Matera A.G. Terns R.M. Terns M.P. (2007) Non-coding RNAs: lessons from the small nuclear and small nucleolar RNAs. Nat. Rev. Mol. Cell Biol., 8, 209–220. 17318225 10.1038/nrm2124

[27] Bartel D.P. (2004) MicroRNAs: genomics, biogenesis, mechanism, and function. Cell, 116, 281–297. 14744438 10.1016/s0092-8674(04)00045-5

[28] He L. Hannon G.J. (2004) MicroRNAs: small RNAs with a big role in gene regulation. Nat. Rev. Genet., 5, 522–531. 15211354 10.1038/nrg1379

[29] Mendell J.T. (2005) MicroRNAs: critical regulators of development, cellular physiology and malignancy. Cell Cycle, 4, 1179–1184. 16096373 10.4161/cc.4.9.2032

[30] Friedman R.C. Farh K.K.H. Burge C.B (2009) Most mammalian mRNAs are conserved targets of microRNAs. Genome Res., 19, 92–105. 18955434 10.1101/gr.082701.108 PMC2612969

[31] Lewis B.P. Burge C.B. Bartel D.P. (2005) Conserved seed pairing, often flanked by adenosines, indicates that thousands of human genes are microRNA targets. Cell, 120, 15–20. 15652477 10.1016/j.cell.2004.12.035

[32] Eddy S.R. (2002) Computational Genomics of Noncoding RNA Genes. Cell, 109, 137–140. 12007398 10.1016/s0092-8674(02)00727-4

[33] Weber M.J. (2006) Mammalian small nucleolar RNAs are mobile genetic elements. PLoS Genet., 2, e205 17154719 10.1371/journal.pgen.0020205 PMC1687206

[34] Schmitz J. Zemann A. Churakov G (2008) Retroposed SNOfall–A mammalian-wide comparison of platypus snoRNAs. Genome Res., 18, 1005–1010. 18463303 10.1101/gr.7177908 PMC2413151

[35] Shao P. Yang J.H. Zhou H (2009) Genome-wide analysis of chicken snoRNAs provides unique implications for the evolution of vertebrate snoRNAs. BMC Genomics, 10, 86 19232134 10.1186/1471-2164-10-86 PMC2653536

[36] Meunier J. Lemoine F. Soumillon M (2013) Birth and expression evolution of mammalian microRNA genes. Genome Res., 23, 34–45. 23034410 10.1101/gr.140269.112 PMC3530682

[37] Huerta-Cepas J. Dopazo H. Dopazo J (2007) The human phylome. Genome Biol., 8, R109 17567924 10.1186/gb-2007-8-6-r109 PMC2394744

[38] Huerta-Cepas J. Marcet-Houben M. Pignatelli M (2010) The pea aphid phylome: a complete catalogue of evolutionary histories and arthropod orthology and paralogy relationships for *Acyrthosiphon pisum* genes. Insect Mol. Biol., 19 (Suppl 2), 13–21. 20482636 10.1111/j.1365-2583.2009.00947.x

[39] Thomas P.D. Kejariwal A. Campbell M.J (2003) PANTHER: a browsable database of gene products organized by biological function, using curated protein family and subfamily classification. Nucleic Acids Res., 31, 334–341. 12520017 10.1093/nar/gkg115 PMC165562

[40] Vilella A.J. Severin J. Ureta-Vidal A (2009) EnsemblCompara GeneTrees: Complete, duplication-aware phylogenetic trees in vertebrates. Genome Res., 19, 327–335. 19029536 10.1101/gr.073585.107 PMC2652215

[41] Fitch W.M. (1970) Distinguishing homologous from analogous proteins. Syst. Zool., 19, 99–113. 5449325

[42] Huerta-Cepas J. Capella-Gutiérrez S. Pryszcz L.P (2014) PhylomeDB v4: zooming into the plurality of evolutionary histories of a genome. Nucleic Acids Res., 42, D897–D902. 24275491 10.1093/nar/gkt1177 PMC3964985

[43] Mi H. Muruganujan A. Thomas P.D. (2013) PANTHER in 2013: modeling the evolution of gene function, and other gene attributes, in the context of phylogenetic trees. Nucleic Acids Res., 41, D377–D386. 23193289 10.1093/nar/gks1118 PMC3531194

[44] Herrero J. Muffato M. Beal K (2011) Ensembl comparative genomics resources. Database (Oxford), 2016, bav096. 10.1093/database/bav096 PMC4761110 26896847

[45] Schreiber F. Patricio M. Muffato M (2014) TreeFam v9: a new website, more species and orthology-on-the-fly. Nucleic Acids Res., 42, D922–D925. 24194607 10.1093/nar/gkt1055 PMC3965059

[46] Hoeppner M.P. White S. Jeffares D.C (2009) Evolutionarily stable association of intronic snoRNAs and microRNAs with their host genes. Genome Biol. Evol., 1, 420–428. 20333211 10.1093/gbe/evp045 PMC2817437

[47] Nawrocki E.P. Eddy S.R. (2013) Infernal 1.1: 100-fold faster RNA homology searches. Bioinformatics, 29, 2933–2935. 24008419 10.1093/bioinformatics/btt509 PMC3810854

[48] Nawrocki E.P. Burge S.W. Bateman A (2015) Rfam 12.0: updates to the RNA families database. Nucleic Acids Res., 43, D130–D137. 25392425 10.1093/nar/gku1063 PMC4383904

[49] Kozomara A. Griffiths-Jones S. (2014) miRBase: annotating high confidence microRNAs using deep sequencing data. Nucleic Acids Res., 42, D68–D73. 24275495 10.1093/nar/gkt1181 PMC3965103

[50] Eddy S.R. Durbin R. (1994) RNA sequence analysis using covariance models. Nucleic Acids Res., 22, 2079–2088. 8029015 10.1093/nar/22.11.2079 PMC308124

[51] Krogh A. Brown M. Mian I.S (1994) Hidden Markov models in computational biology. Applications to protein modeling. J. Mol. Biol., 235, 1501–1531. 8107089 10.1006/jmbi.1994.1104

[52] Gardner P.P. (2009) The use of covariance models to annotate RNAs in whole genomes. Brief. Funct. Genomic. Proteomic., 8, 444–450. 19833700 10.1093/bfgp/elp042

[53] Jow H. Hudelot C. Rattray M (2002) Bayesian phylogenetics using an RNA substitution model applied to early mammalian evolution. Mol. Biol. Evol., 19, 1591–1601. 12200486 10.1093/oxfordjournals.molbev.a004221

[54] Iwama H. Kato K. Imachi H (2013) Human microRNAs originated from two periods at accelerated rates in mammalian evolution. Mol. Biol. Evol., 30, 613–626. 23171859 10.1093/molbev/mss262 PMC3563971

[55] Severin J. Beal K. Vilella A (2010) eHive: An Artificial Intelligence workflow system for genomic analysis. BMC Bioinformatics, 11, 240 20459813 10.1186/1471-2105-11-240 PMC2885371

[56] Bininda-Emonds O.R.P. (2004) The evolution of supertrees. Trends Ecol. Evol., 19, 315–322. 16701277 10.1016/j.tree.2004.03.015

[57] Howe K. Bateman A. Durbin R. (2002) QuickTree: building huge Neighbour-Joining trees of protein sequences. Bioinformatics, 18, 1546–1547. 12424131 10.1093/bioinformatics/18.11.1546

[58] Löytynoja A. Goldman N. (2008) Phylogeny-Aware Gap Placement Prevents Errors in Sequence Alignment and Evolutionary Analysis. Science (80-.)., 320, 1632–1635. 10.1126/science.1158395 18566285

[59] Löytynoja A. Goldman N. (2010) webPRANK: a phylogeny-aware multiple sequence aligner with interactive alignment browser. BMC Bioinformatics, 11, 579 21110866 10.1186/1471-2105-11-579 PMC3009689

[60] Soria-Carrasco V. Talavera G. Igea J (2007) The K tree score: quantification of differences in the relative branch length and topology of phylogenetic trees. Bioinformatics, 23, 2954–2956. 17890735 10.1093/bioinformatics/btm466

[61] De Bie T. Cristianini N. Demuth J.P (2006) CAFE: a computational tool for the study of gene family evolution. Bioinformatics, 22, 1269–1271. 16543274 10.1093/bioinformatics/btl097

[62] Hahn M.W. Demuth J.P. Han S.G. (2007) Accelerated rate of gene gain and loss in primates. Genetics, 177, 1941–1949. 17947411 10.1534/genetics.107.080077 PMC2147951

[63] Kim Y.K. Kim V.N. (2007) Processing of intronic microRNAs. Embo J., 26, 775–783. 17255951 10.1038/sj.emboj.7601512 PMC1794378

[64] Hsu S.D. Chu C.H. Tsou A.P (2008) miRNAMap 2.0: genomic maps of microRNAs in metazoan genomes. Nucleic Acids Res., 36, D165–D169. 18029362 10.1093/nar/gkm1012 PMC2238982

[65] Han M.V. Zmasek C.M. (2009) phyloXML: XML for evolutionary biology and comparative genomics. BMC Bioinformatics, 10, 356 19860910 10.1186/1471-2105-10-356 PMC2774328

[66] Schmitt T. Messina D.N. Schreiber F (2011) SeqXML and OrthoXML: standards for sequence and orthology information. Brief. Bioinform., 12, 485–488. 21666252 10.1093/bib/bbr025

[67] Bateman A. Agrawal S. Birney E (2011) RNAcentral: A vision for an international database of RNA sequences. Rna, 17, 1941–1946. 21940779 10.1261/rna.2750811 PMC3198587

[68] Gerlach D. Kriventseva E.V. Rahman N (2009) miROrtho: computational survey of microRNA genes. Nucleic Acids Res., 37, D111–D117. 18927110 10.1093/nar/gkn707 PMC2686488

[69] Librado P. Vieira F.G. Rozas J. (2012) BadiRate: estimating family turnover rates by likelihood-based methods. Bioinformatics, 28, 279–281. 22080468 10.1093/bioinformatics/btr623

[70] Csurös M. (2010) Count: evolutionary analysis of phylogenetic profiles with parsimony and likelihood. Bioinformatics, 26, 1910–1912. 20551134 10.1093/bioinformatics/btq315

[71] Burge S.W. Daub J. Eberhardt R (2013) Rfam 11.0: 10 years of RNA families. Nucleic Acids Res., 41, D226–D232. 23125362 10.1093/nar/gks1005 PMC3531072

[72] Zuker M. Stiegler P. (1981) Optimal computer folding of large RNA sequences using thermodynamics and auxiliary information. Nucleic Acids Res., 9, 133–148. 6163133 10.1093/nar/9.1.133 PMC326673

[73] Lowe T.M. Eddy S.R. (1997) tRNAscan-SE: a program for improved detection of transfer RNA genes in genomic sequence. Nucleic Acids Res., 25, 955–964. 9023104 10.1093/nar/25.5.955 PMC146525

[74] Guttman M. Amit I. Garber M (2009) Chromatin signature reveals over a thousand highly conserved large non-coding RNAs in mammals. Nature, 458, 223–227. 19182780 10.1038/nature07672 PMC2754849

[75] Stamatakis A. (2006) RAxML-VI-HPC: maximum likelihood-based phylogenetic analyses with thousands of taxa and mixed models. Bioinformatics, 22, 2688–2690. 16928733 10.1093/bioinformatics/btl446

[76] Hudelot C. Gowri-Shankar V. Jow H (2003) RNA-based phylogenetic methods: application to mammalian mitochondrial RNA sequences. Mol. Phylogenet. Evol., 28, 241–252. 12878461 10.1016/s1055-7903(03)00061-7

[77] Price M.N. Dehal P.S. Arkin A.P. (2010) FastTree 2–approximately maximum-likelihood trees for large alignments. PLoS One, 5, e9490. 20224823 10.1371/journal.pone.0009490 PMC2835736

[78] Stamatakis A. Aberer A.J. Goll C (2012) RAxML-Light: a tool for computing terabyte phylogenies. Bioinformatics, 28, 2064–2066. 22628519 10.1093/bioinformatics/bts309 PMC3400957

[79] Sayers E.W. Barrett T. Benson D.A (2012) Database resources of the National Center for Biotechnology Information. Nucleic Acids Res., 40, D13–D25. 22140104 10.1093/nar/gkr1184 PMC3245031

[80] Hedges S.B. Dudley J. Kumar S. (2006) TimeTree: a public knowledge-base of divergence times among organisms. Bioinformatics, 22, 2971–2972. 17021158 10.1093/bioinformatics/btl505

