# Supplementary material for: ncRNA orthologies in the vertebrate lineage
Source: Database (Oxford). 2016 Mar 15;2016:bav127. doi: 10.1093/database/bav127 (PMC4792531; doi:10.1093/database/bav127)
Supplement: Supplementary Data [file supp_2016_bav127_index.html]

ncRNA orthologies in the vertebrate lineage — Supplementary Data 

# ncRNA orthologies in the vertebrate lineage

## Supplementary Data

files

- Supplementary Data - png file
