# Supplementary figures and images for: ncRNA orthologies in the vertebrate lineage
Source: Database (Oxford). 2016 Mar 15;2016:bav127. doi: 10.1093/database/bav127 (PMC4792531; doi:10.1093/database/bav127)

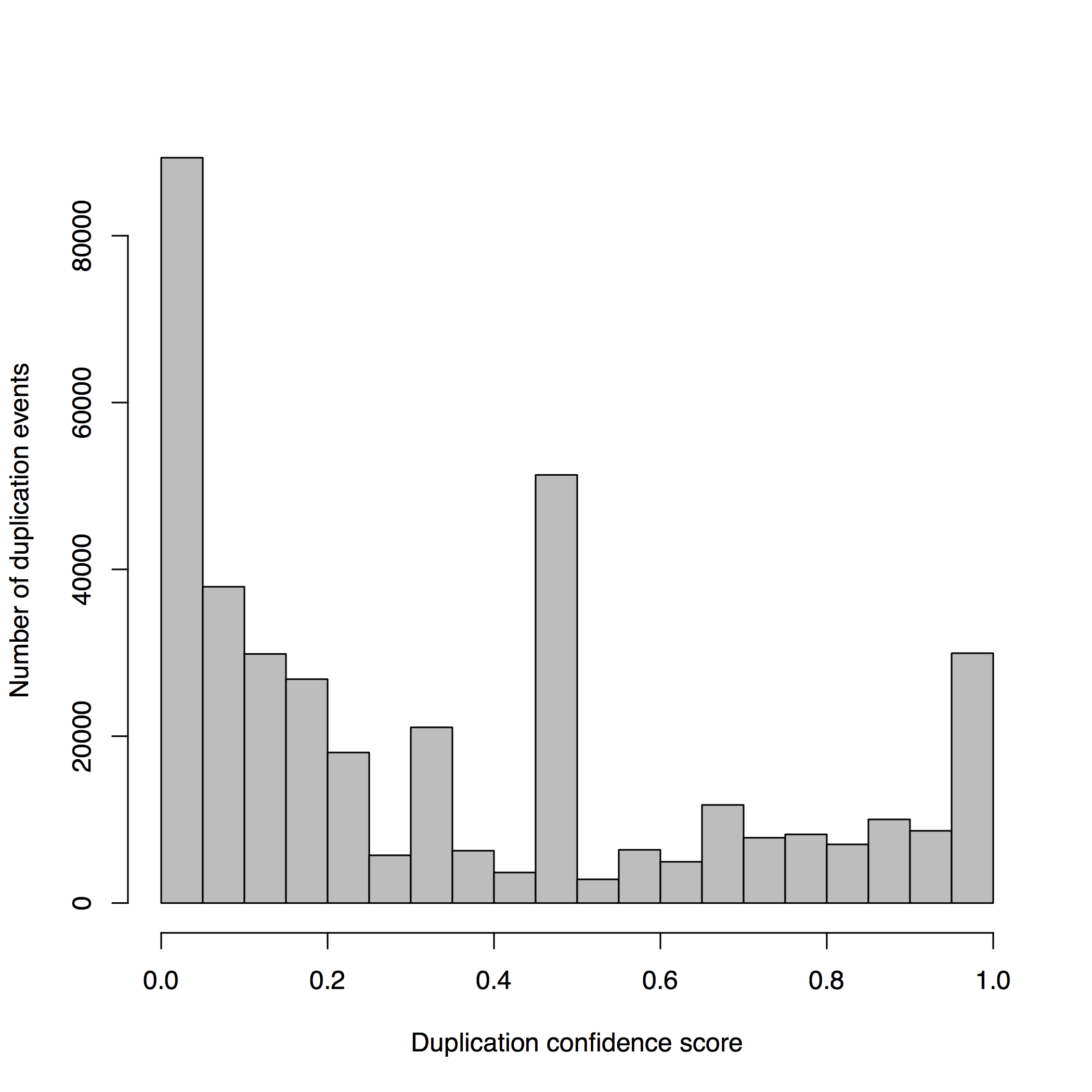

Supplement: Supplementary Data [file supp_bav127_SuppFig1_e82.png]
